# A New Method for Dispersing Pristine Carbon Nanotubes Using Regularly Arranged S-Layer Proteins

**DOI:** 10.3390/nano11051346

**Published:** 2021-05-20

**Authors:** Andreas Breitwieser, Uwe B. Sleytr, Dietmar Pum

**Affiliations:** 1 Department of Nanobiotechnology, Institute of Biophysics, University of Natural Resources and Life Sciences Vienna, 1190 Vienna, Austria; andreas.breitwieser@boku.ac.at; 2 Department of Nanobiotechnology, Institute of Synthetic Bioarchitectures, University of Natural Resources and Life Sciences Vienna, 1190 Vienna, Austria; uwe.sleytr@boku.ac.at

**Keywords:** S-layer protein, pristine carbon nanotubes, graphene, dispersion, non-covalent functionalization, aqueous solution, biomineralization

## Abstract

Homogeneous and stable dispersions of functionalized carbon nanotubes (CNTs) in aqueous solutions are imperative for a wide range of applications, especially in life and medical sciences. Various covalent and non-covalent approaches were published to separate the bundles into individual tubes. In this context, this work demonstrates the non-covalent modification and dispersion of pristine multi-walled carbon nanotubes (MWNTs) using two S-layer proteins, namely, SbpA from *Lysinibacillus sphaericus* CCM2177 and SbsB from *Geobacillus stearothermophilus* PV72/p2. Both the S-layer proteins coated the MWNTs completely. Furthermore, it was shown that SbpA can form caps at the ends of MWNTs. Reassembly experiments involving a mixture of both S-layer proteins in the same solution showed that the MWNTs were primarily coated with SbsB, whereas SbpA formed self-assembled layers. The dispersibility of the pristine nanotubes coated with SbpA was determined by zeta potential measurements (−24.4 +/− 0.6 mV, pH = 7). Finally, the SbpA-coated MWNTs were silicified with tetramethoxysilane (TMOS) using a mild biogenic approach. As expected, the thickness of the silica layer could be controlled by the reaction time and was 6.3 +/− 1.25 nm after 5 min and 25.0 +/− 5.9 nm after 15 min. Since S-layer proteins have already demonstrated their capability to bind (bio)molecules in dense packing or to act as catalytic sites in biomineralization processes, the successful coating of pristine MWNTs has great potential in the development of new materials, such as biosensor architectures.

## 1. Introduction

In recent decades, research efforts have focused on the development of organic–inorganic hybrid nanomaterials, as they were expected to have excellent physical and (bio)chemical properties for developments in the materials and life sciences [1,2]. Carbon nanotubes (CNTs), fullerenes, and graphene are among the most promising candidates for such emerging technologies [3,4]. In addition to traditional approaches, bioinspired strategies using proteins and peptides have attracted much attention, as these biomolecules have been optimized over billions of years of evolution for specific functions. In particular, the biomedical applications of CNTs have increased tremendously in recent years, such as cellular imaging or drug-targeting and delivery in cancer therapies [5,6,7]. However, further progress in all these applications is only possible when homogeneous and stable dispersions of functionalized CNTs in aqueous solutions are available. The tendency of carbon nanotubes to form bundles and, eventually, to become insoluble in water is caused by their strong hydrophobic and cohesive van der Waals interactions. In order to overcome this problem, several approaches have been developed to separate bundles into individual tubes. The covalent functionalization of surface chemical groups, such as the oxidation of carbon, and non-covalent functionalization by attaching amphiphilic molecules, such as sodium dodecyl sulfate (SDS), have proven to disperse CNT aggregates successfully [8,9,10,11]. Nevertheless, the non-covalent modification of carbon nanotubes with functional proteins, which are also able to bind further (bio)molecules or act as catalytic sites in biomineralization processes [12], is highly desirable, since this approach will lay the foundation for a carbon nanotube-based biomolecular construction kit. In this context, we have previously shown that wild-type and genetically modified SbpA S-layer proteins from *Lysinibacillus sphaericus* CCM2177 (identical to *L. sphaericus* ATCC 4525) [13,14] are able to completely coat oxidized multi-walled carbon nanotubes (MWNTs) in a helical crystalline arrangement and allow for the specific binding of further biomolecules, such as immunoglobulin G (IgG) [15]. However, coating pristine CNTs also seemed very attractive to us because CNTs do not have to be oxidized beforehand, and thus, with respect to covalent functionalization, the integrity of the (electronic) structure of the CNTs is preserved [16].

**Figure 1:**
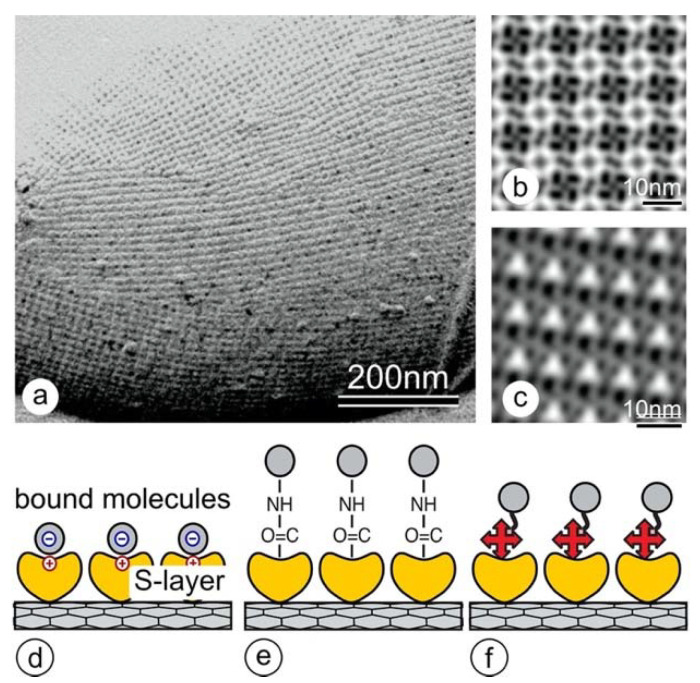
(**a**) TEM micrograph of a freeze-etched and metal shadowed preparation of the cell surface of *Lysinibacillus sphaericus* CCM 2177. The S-layer, which is the outermost cell wall component, shows a square lattice symmetry. The numerous lattice defects are necessary for the S-layer lattice to cover the curved cell pole. In the 2D-image reconstructions, the square (p4) lattice symmetry of the SbpA (**b**) and the oblique (p1) of the SbsB (**c**) become clearly visible. The protein is bright, while the staining in the pores and wells is dark. (**d**–**f**) Schematic drawings of S-layer protein arrays used for binding (bio)molecules by (**d**) electrostatic interactions, (**e**) surface chemical groups (e.g., after carbodiimide activation of carboxyl groups), or (**f**) functional domains in S-layer fusion proteins (e.g., by making use of the streptavidin-biotin interaction).

S-layer protein lattices (also termed S-layers) are one of the most common cell surface structures in bacteria and archaea and completely cover the cells (Figure 1a) [17,18]. S-layer proteins (M_w_ 40–200 kDa) are strain-specific and have the natural capability to form regular arrays not only on the cell surface but also in solutions, on lipid layers, and solid supports by self-assembly [17,19]. Unit cell sizes range from 3 to 30 nm and thicknesses from 5 to 10 nm (up to 70 nm in archaea). Depending on the lattice type, which may be either oblique (p1, p2), square (p4), or hexagonal (p3, p6), a single unit cell contains either one, two, four, three, or six identical S-layer proteins, respectively. S-layer lattices are isoporous protein meshes, with pore sizes in the ultrafiltration range of 2 to 8 nm [20]. The outer face of the S-layer is usually flat, while the inner one is corrugated [17]. The surface charge of the two faces is determined by the amount of free carboxyl- and amino groups. Accordingly, the outer face is charge-neutral, and the inner one is either net positively or negatively charged.

In addition, S-layers have been used not only as binding and affinity matrices for biomolecules (Figure 1d–f) [17,21,22] but also as activation layers for the biogenic mineralization of silica [23,24] or the formation of metallic nanoparticle arrays [25,26,27,28,29]. One of these developments was our work on silica-reinforced S-layer protein cages, where mechanically stable, hollow, and porous silica spheres were obtained after the removal of the lipid core [24]. 

In this work, we introduce a new facile protocol for the non-covalent functionalization and dispersion of MWNTs with two different S-layer proteins, namely, SbpA from *L. sphaericus* CCM2177 (Figure 1a,b) [13] and SbsB from *Geobacillus stearothermophilus* PV72/p2 (Figure 1c) [30]. We demonstrate their impact on the dispersibility of pristine carbon nanotubes in an aqueous buffer, their complete coating, and careful biogenic silicification of SbpA, leading to silica-coated nanotubes with controlled wall thicknesses.

## 2. Materials and Methods

### 2.1. Production of Wild-Type and Recombinant S-Layer Protein Solutions

*L. sphaericus* CCM 2177 was grown in a continuous culture, as described in a previous study [31]. After a downstream process, the purified cell wall fragments were extracted with 5 M guanidine hydrochloride (GHCL, Gerbu Nr. 1057) and, after centrifugation steps, dialyzed (membrane Biomol cut-off: 12–16 kD; pore size 2.5 nm) against 3 L Milli-Q water containing 2 mM EDTA. The produced monomeric wild-type SbpA (wtSbpA) S-layer protein solution was adjusted to a final concentration of 1 mg/mL. 

The recombinant S-layer (rSbpA) protein was expressed in *E. coli*, as described previously [13]. rSbpA accumulated in inclusion body-like structures, from which the S-layer protein was extracted and subjected to gel permeation chromatography (GPC) using a Superdex 200 column (Cytiva) and 2 M guanidine hydrochloride (GHCl) in a 50 mM Tris-HCl buffer (pH 7.2) as the extraction and running buffer. The fractions containing the target protein were pooled and dialyzed against Milli-Q water. Again, the concentration was adjusted to 1 mg/mL. 

To produce biomass containing the bacterial cells coated with an S-layer, *Geobacillus stearothermophilus* PV72/p2 was grown in a batch culture. After a purification process using wet biomass, the S-layer protein, SbsB, was extracted, dialyzed, and adjusted to 1 mg/mL, as described in [32]. In contrast to the protein solutions based on the S-layer proteins from SbpA (wt and rSbpA), which can be stored over a longer period of time, since their reassembly depends on the addition of CaCl_2_ ions, the protein solution of SbsB was produced immediately before the coating experiments.

### 2.2. Coating of Pristine and Oxidized Multi-Walled Carbon Nanotubes (MWNTs)

For almost all coating experiments, the MWNTs from SIGMA (Saint Louis, MO, USA; diameter of 50–90 nm, Nr. 901019) were used in their pristine form or after oxidation by introducing −COOH groups, as described in [15]. Only for co-crystallization experiments with wtSbpA and SbsB were oxidized MWNTs used with diameters ranging from 110 to 170 nm (SIGMA; Saint Louis, MO, USA; Nr. 659258). The coating protocol was optimized, which allowed for not only a more accurate determination of the ratio of MWNTs to applied S-layer protein but also the coating of pristine nanotubes. Therefore, 3 mg of nanotubes was resuspended in 30 mL phosphate-buffered saline (PBS) containing 0.1% Triton X 100 (SIGMA, Saint Louis, MO, USA; Nr. T9284) and dispersed using ultrasonication (Branson Sonifier 250; output 5, duty circle 50%) for 20 min. The so-treated nanotubes could be stored for further coating experiments for at least 6 months at 4 °C.

For the actual coating with S-layer proteins, 4 mL of the MWNTs stored in PBS/Triton were centrifuged (Eppendorf; Centrifuge 5424, Hamburg, Germany) at 5000 rcf for 10 min, re-suspended in a 4.8 mL crystallization buffer (5 mM Tris buffer with 0.1 M CaCl_2,_ pH = 9.0), followed by a sonification step for 2 min in an ice bath. Immediately, 200 µL of the selected S-layer protein solution (1 mg/mL) was added, and ultrasonication in the ice bath was prolonged for 4 min. Then, recrystallization was allowed to take place at 4 °C overnight. These dispersions were stable at 4 °C for a minimum of 6 months.

### 2.3. Coating of Graphene Sheets with S-Layer Protein SbpA

The reassembly of wtSbpA on flat, solid-supported graphene sheets (Nanografi, Turkey, Ankara, NG01GS0104) was studied as well. Slabs (0.7 cm × 0.7 cm) were cut out of a graphene sheet and washed with PBS/Triton and Milli-Q water. After air drying, the slabs were mounted on silicon wafers and incubated with wtSbpA (100 µg/mL crystallization buffer containing CaCl_2_) overnight. After being washed with Milli-Q water, the coated slabs were examined with an atomic force microscope.

### 2.4. Silicification of SbpA-Coated MWNTs

The S-layer-coated pristine nanotubes were allowed to adsorb on 300-mesh carbon-coated copper grids (Agar Scientific Ltd., Stansted, UK) for 30 min. Subsequently, the grids were washed in Milli water, and silicification was performed, as described in [23]. For the deposition and biomineralization of the S-layer coated MWNTs, tetramethoxysilane (TMOS) was used. A solution of silicic acid was freshly prepared by dissolving Si(OCH_3_)_4_ (TMOS, Sigma–Aldrich) in 1 mM HCl to a final concentration of 1 M (hydrolysis). A phosphate buffer (K_2_HPO_4_/KH_2_PO_4_; pH 7.2) was added to obtain a final concentration of 0.1 M TMOS, and silicification was allowed to take place for 5, 15, 30, and 60 min. Subsequently, the grids were washed with Milli-Q water and investigated, without further staining, in a transmission electron microscope.

### 2.5. Transmission Electron Microscopy (TEM)

Transmission electron microscopy with an FEI Tecnai T20 G^2^ operated at 160 kV (FEI Europe (now ThermoScientific), Eindhoven, The Netherlands) was used to control and take images of the successful S-layer coating of MWNTs and the deposited thin silica layers in the silica (S-layer) MWNT constructs. For this purpose, samples were adsorbed on 300-mesh carbon-coated copper grids (Agar Scientific Ltd., Stansted, UK). While S-layer MWNT samples were negatively stained by placing them on 2% uranium acetate drops for 10 min, silicified S-layer-coated nanotube samples were not negatively stained in order to obtain contrast in the TEM images from the silica layer only. All steps were performed at room temperature.

### 2.6. Atomic Force Microscopy (AFM)

Atomic force microscopy was performed with a Multimode AFM (Bruker AXS, Santa Barbara, CA, USA) equipped with a Nanoscope-V controller and an E-scanner, with a scan range up to 12 µm. In this study, silicon-nitride probes (MSNL-10, Bruker, Santa Barbara, CA, USA), with a nominal spring constant of 0.2 N/m, were used. The samples were investigated in an aqueous 0.1 M NaCl solution in contact mode by applying low loading forces (<1 nN) to avoid sample damaging, with scan rates of 1–4 Hz. 

### 2.7. Zeta-Potential Measurements of rSbpA-Coated MWNTs

In contrast to uncoated carbon nanotubes, it was possible to determine the zeta potential of S-layer-coated pristine and oxidized MWNTs due to their excellent and homogenous dispersion in buffer systems. The rSbpA-coated MWNTS, produced as described above, were diluted 1:10 with Milli-Q water, and the zeta potential measurements were performed using a Malvern Zetasizer (Nano series, Nano–ZS; Malvern Instruments, Worcestershire, UK).

## 3. Results and Discussion

### 3.1. Reassembly of SbpA and SbsB S-Layer Protein on Pristine MWNTs

The successful reassembly of S-layer proteins on MWNTs is fundamental to all further steps in the development of the new hybrid S-layer, carbon nanotube composites. This step includes their silicification, which is investigated in this work as well. While we have already successfully developed a protocol for the coating of oxidized MWNTs, the focus of the work presented here was the functionalization of pristine CNTs with SbpA S-layer protein from *L. sphaericus* CCM2177 [13] and SbsB S-layer protein from *G. stearothermophilus* PV72/p2 [30]. The two S-layer proteins, SbpA and SbsB, differ in terms of their lattice parameters and lattice symmetry, as shown in Table 1.

The protocol described in [15] allowed for the coating of oxidized MWNTs with different S-layer proteins. In this work, the MWNTs were resuspended in PBS/Triton by ultrasonication, before the actual coating process, which resulted in a well-dispersed homogeneous distribution of the MWNTs in an aqueous solution. The MWNTs, dispersed in this way, not only could be stored for a longer period of time but also allowed for the quantification of the applied S-layer protein concentration in relation to the amount of carbon nanotubes used. In this way, it was now also possible to minimize the formation of S-layer self-assembly products in the solution. Since the S-layer proteins cannot reassemble in the presence of Triton X 100, the detergent had to be washed away in a centrifugation step. Subsequently, the carbon nanotubes were resuspended in a crystallization buffer containing CaCl_2_, and the coating step with the respective S-layer protein followed in due course. Since the carbon nanotubes had the tendency to immediately aggregate again in the crystallization buffer, a further ultrasonication step was performed for 2 min, before adding the S-layer protein. Finally, the ultrasonication was continued for 4 min. Since after 4 min of incubation with S-layer protein, the MWNTs were already finely dispersed, we assumed a rapid attachment of the S-layer proteins to the MWNT surfaces, as expected from the two-step, non-classical pathway of S-layer reassembly [33,34,35]. The final transition to the crystalline state was allowed to occur overnight. To avoid denaturation effects, overheating was suppressed by performing all ultrasonication steps in an ice bath. With this optimized protocol, the pristine MWNTs could also be efficiently coated with highly ordered S-layer protein monolayers.

**Figure 2:**
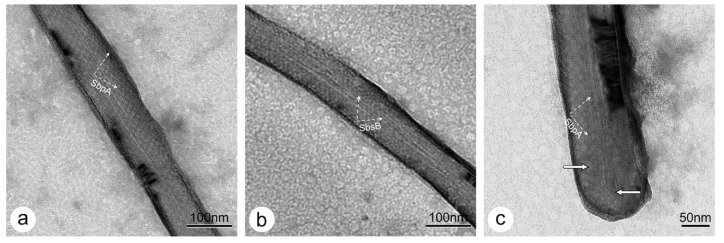
TEM images of negatively stained (**a**) SbpA- and (**b**) SbsB-coated pristine MWNTs. The end of a MWNT closed with a SbpA cap is shown in (**c**). The S-layer lattice shows a good long-range order in the cylindrical part, while lattice defects can be seen close to or on the cap, which is a requirement for the protein lattice to cover the curved surface (marked by arrows).

The diameters of the MWNTs used in this work ranged from 50 to 170 nm. TEM images of negatively stained preparations of pristine MWNTs coated with SbpA and SbsB S-layer protein lattices are shown in Figure 2a,b, respectively.

In this context, we also addressed the question of whether it is possible that the S-layer protein, SbpA, used in this study, forming arrays with a square (p4) lattice symmetry and a lattice spacing of 13.1 nm, can follow the curved, hemispherical surfaces of closed nanotubes (as shown in Figure 2c). We assume that the S-layer proteins model the caps with a certain flexibility in their crystalline arrangement. The dark “shadow” around the cap is probably caused by an accumulation of staining.

**Figure 3:**
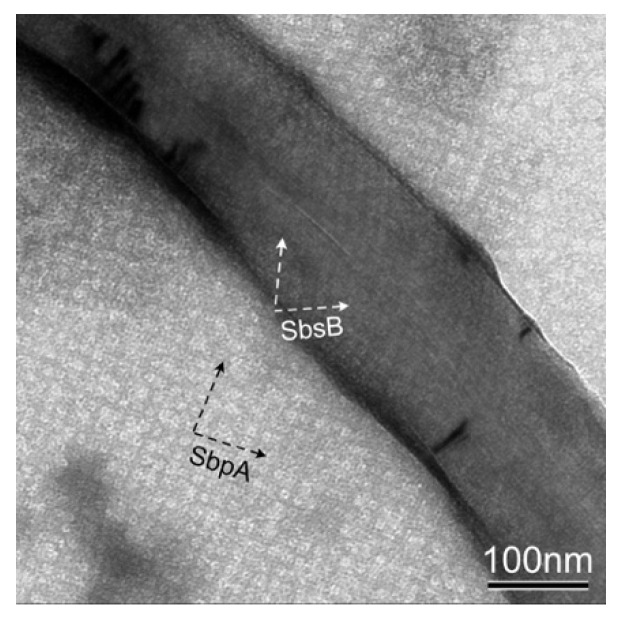
TEM image of the heterologous reassembly of SbpA and SbsB S-layer proteins in a pristine MWNT solution. SbsB covers the nanotube surface (white arrows), while SbpA forms self-assembled sheets (black arrows).

In addition, reassembly experiments, where a mixture of both S-layer proteins, SbpA and SbsB, was added to a solution of pristine MWNT, were carried out (Figure 3) [33]. It is worth noting that the carbon nanotube is covered by SbsB with an oblique lattice symmetry, while SbpA formed self-assembly products with a square lattice symmetry in the background. While it may be assumed that MWNTs covered with both S-layer proteins can be found too, we were not successful in this case [33]. Thus, we assumed that the competition in the formation of a self-assembled layer on the MWNT surface is favored by the simpler oblique (p1) lattice symmetry of SbsB, where only one monomer has to be added to the boundary of the growing lattice, while in the square (p4) lattice of SbpA, four monomers have to find their correct position and orientation first, before the assembly of the next unit cell begins [34]. In this context, it also seems plausible that it is easier for the simple oblique (p1) lattice of SbsB to follow the curvature of the nanotubes than for the probably stiffer higher symmetric square (p4) lattice of SbpA [35]. The concurrent self-assembly of both S-layer proteins also includes an intrinsic self-purification process, since for each of the two S-layer proteins, the other one is an impurity [36].

### 3.2. Reassembly of SbpA S-Layer Protein on Graphene

**Figure 4:**
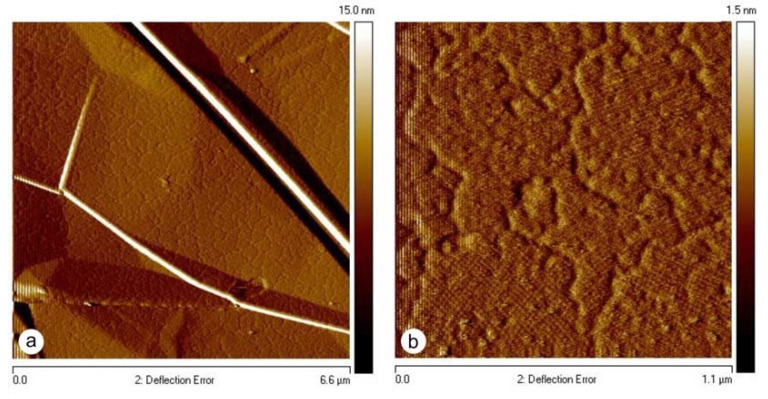
AFM images of a mosaic of crystalline SbpA S-layer domains on graphene. (**a**) An overview, (**b**) a zoomed view.

Moreover, the developed preparation protocol for coating pristine MWNTs could also be successfully applied for the self-assembly of SbpA S-layer protein on graphene (Figure 4). After the injection of the S-layer protein into the solution containing the graphene sheets, an S-layer protein monolayer started to grow in a non-classical pathway from randomly distributed nucleation sites on the graphene surface and continued until the front lines of the individually growing domains met. In this way, an extended mosaic of monocrystalline areas was formed, as demonstrated already for the reassembly on solid supports, such as silicon and mica, or on solid-supported lipid bilayers [37,38,39]. The functionalization of graphene with 2D-crystalline monomolecular S-layer protein lattices is completely new and allows graphene to be endowed with bio-specific functionalities when S-layer fusion proteins are used. Examples would be the binding domains for antibodies or streptavidin for biotinylated molecules (Figure 1f) [15,40].

### 3.3. Zeta-Potential Measurements of SbpA S-Layer Protein-Coated MWNTs

When S-layer protein was added and ultrasonication was prolonged, the suspension became immediately homogeneous. The dispersed suspension was then stable for several months and only had to be shaken in order to resuspend the sedimented S-layer-coated MWNTs again. The stability of the dispersion depends on the balance between the repulsive electrostatic charges on the S-layer-coated carbon nanotubes and the van der Waals attraction. Thus, zeta-potential measurements were performed in order to characterize the dispersibility of S-layer-coated MWNTs [10,11,41,42]. It was assumed that the amphiphilic S-layer proteins are instantaneously attached to the MWNT surface and, in this way, shield the highly hydrophobic and almost charge-neutral MWNT surfaces from the aqueous medium. Zeta-potential measurements of SbpA-coated pristine MWNTs yielded −24.4 +/− 0.6 mV (pH = 7). The isoelectric point of SbpA is at pH = 4.6 [43]. For the sake of completeness, the zeta-potential of SbpA S-layer-coated oxidized MWNTs was −30.1 mV +/− 0.5 mV. The zeta-potential of uncoated pristine MWNTs could not be determined due to their strong agglomeration in an aqueous solution.

### 3.4. Silicification of SbpA S-Layer Protein-Coated MWNTs

**Figure 5:**
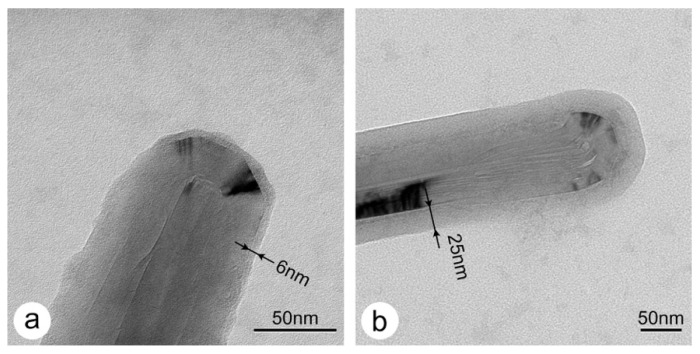
TEM images of silicified SbpA S-layer-coated MWNTs. (**a**) after 5 min, and (**b**) after 15 min silicification time.

In a previous work, S-layer proteins were used as scaffolds for making hybrid organic–inorganic nanostructures, such as silicified S-layer sheets [23] and S-layer coated liposomes [24]. Based on this approach and selected protocols from the literature [12,44], the silicification of S-layer-coated MWNTs with TMOS was investigated in this work too. The thicknesses of the silica layers were determined after 5 min with 6.3 +/− 1.25 nm (n = 24) and after 15 min with 25.0 +/− 5.9 nm (n = 24). TEM images of SbpA-coated MWNTs with ca. 6 nm- and ca. 25 nm-thick amorphous silica layers are shown in Figure 5a,b, respectively. It is worth noting that the silica thickness after 5 min of silicification is close to the thickness of the S-layer and might resemble the isoporous S-layer ultrastructure. However, although the contrast in these images originates from the silica only, the silica shell is not able to show the molecular structure of the S-layer, as compared to negative staining with uranyl acetate. Nevertheless, the typical graphitic type fringes in the TEM images of MWNTs are visible. No results could be obtained for 30 min and 60 min of silicification, since the EM-grids had been completely covered by a thick amorphous silica layer. It should also be emphasized that biogenic silicification under mild aqueous conditions was only possible when the MWNTs had previously been dispersed by the S-layer proteins.

## 4. Conclusions

The development of an enabling technology for making functionalized and biomineralized nanotubes based on the self-assembly of S-layer proteins is completely new in the field of biomaterial research [15]. Moreover, it is not only the coating of the nanotube surface or graphene with an (often randomly arranged) biopolymer [45,46,47,48,49], but also the complete coverage with a molecularly precisely defined protein lattice of the same thickness and the repetitive arrangement of functional groups or domains (Figure 1d–f) that are important for all further developments. For example, we already showed that S-layer fusion proteins allow for a highly specific and sensitive functionalization of surfaces in the development of affinity matrices and biosensor surfaces [17,21]. Examples include the IgG-binding domain [50,51,52], the Bet-v1 domain specific for the major birch pollen allergen [53] or for a wide range of applications, the biotin-binding domain [40], and the affinity tag for streptavidin [54] (see [17] for a full list of S-layer fusion proteins and their potential applications). Moreover, a dense layer of S-layer-coated CNTs on native, chemically modified, or even S-layer-coated solid supports allow for a significant increase in exposed surface area. As an example, the introduction of thiol (SH)-groups on gold-coated supports enable the development of novel functional sensor layers. Based on our experience in fabricating multi-enzyme amperometric biosensors [55], it is possible to develop stoichiometrically well-adjusted sensor layers, possibly even using a layer-by-layer (LbL) technique [49]. Furthermore, especially for the development of biomedical devices, the biocompatibility and antifouling properties of S-layer-coated surfaces are worth mentioning [43].

The proposed methodology for making biogenic silica nanotubes based on S-layers is new and has promising applications. However, in particular, we would also like to anticipate that, in line with our work on silicified S-layer-coated liposomes, very thin silica coatings that resemble the isoporous S-layer mesh will become possible and thus could find new applications as novel nanocontainers [56]. In summary, the application potential of these novel composites could be great, especially considering the high and specific binding capacity of S-layer fusion proteins for additional biomolecules.

## Figures and Tables

**Table 1:** Summary of the lattice parameters of the S-layer proteins, SbpA and SbsB, used in this work.

S-Layer Protein	Bacterial Strain	S-Layer Lattice Symmetry	Lattice Parameters
SbpA	*L. sphaericus* CCM2177	square (p4)	a = b = 13.1 nm, d = 9 nm base angle γ = 90°
SbsB	*G. stearothermophilus* PV72/p2	oblique (p1)	a = 10.4 nm, b = 7.9 nm, d = 4.5 nm base angle γ = 81°

## Data Availability

Not applicable.
